# Supplementary material for: The Sperm Protein Spaca6 is Essential for Fertilization in Zebrafish
Source: Front Cell Dev Biol. 2022 Jan 3;9:806982. doi: 10.3389/fcell.2021.806982 (PMC8762341; doi:10.3389/fcell.2021.806982)
Supplement: Supplementary file 2 [file DataSheet1.PDF]

Supplementary Material

1 Supplementary Figures

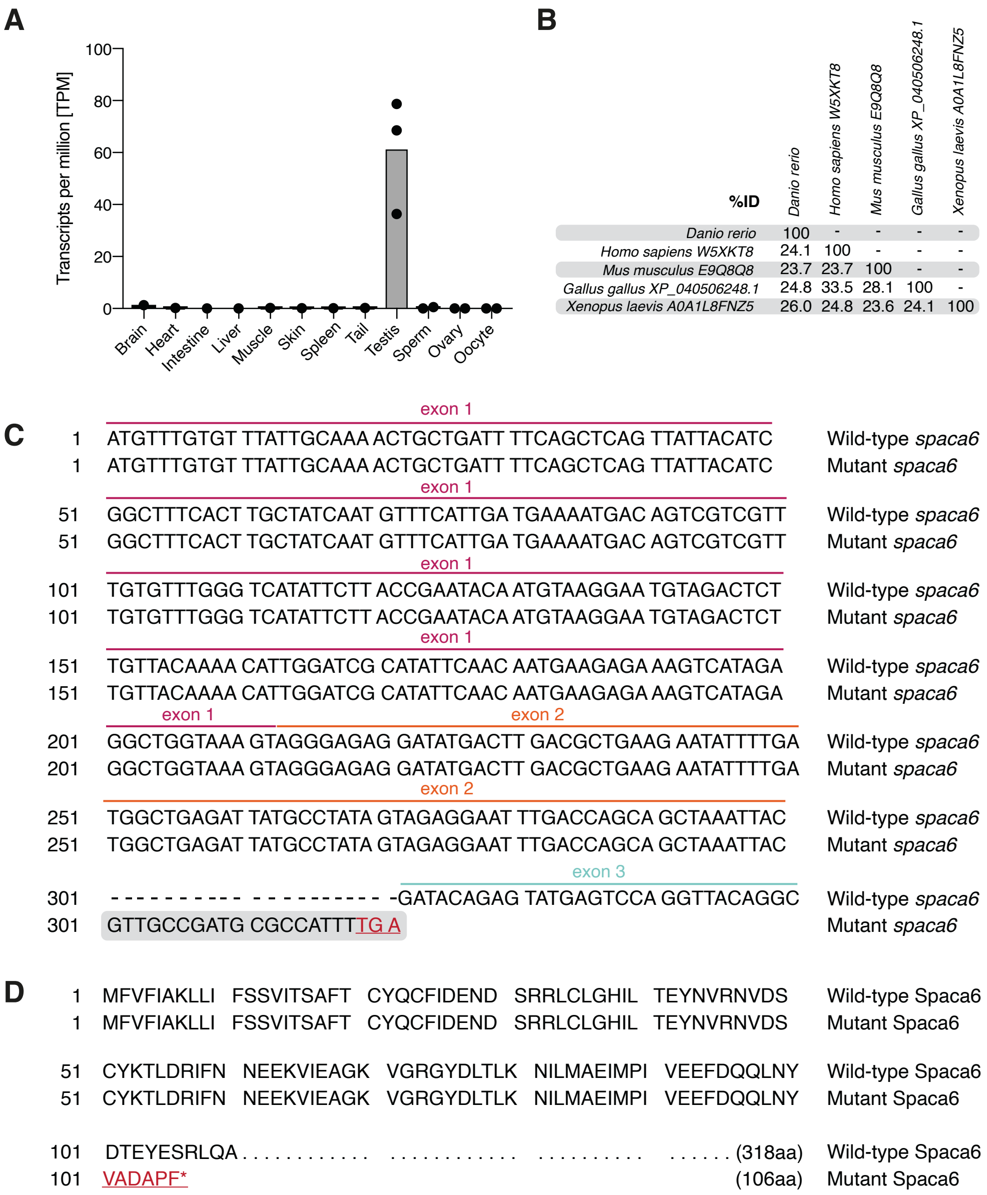

Supplementary Figure S1. Characterization of *spaca6* in zebrafish. A. RNA-Seq analysis of *spaca6* gene expression levels in various adult tissues. Gray bar, mean. TPM, transcripts per million. **B.** Percentage of protein identity of mature Spaca6 protein between different vertebrate species. **C.** Partial cDNA sequence alignment of wild-type and mutant *spaca6*. The first 329 base pairs of the wild-type cDNA sequence were aligned to the mutant cDNA sequence, which retains part of intron 2 (shaded in gray) and contains a stop codon (red, underlined). **D.** Partial amino acid sequence alignment for wild-type (amino acids 1-110) and mutant (full-length) Spaca6. Retained intronic sequence in mutant *spaca6* leads to the translation of 6 additional amino acids before a premature stop codon (red, underlined).

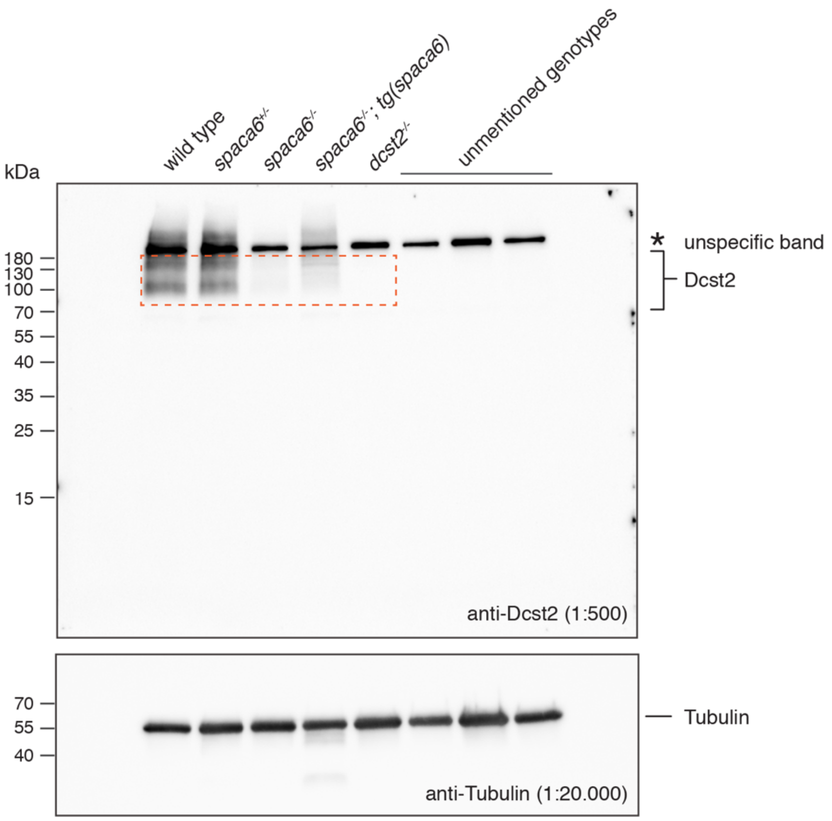

Supplementary Figure S2. Dcst2 levels are reduced in zebrafish *spaca6* KO sperm. Uncropped immunoblot of sperm samples probed with antibodies against zebrafish Dcst2. An unspecific band (asterisk) is detected in all genotypes above 180 kDa. The cropped region is highlighted by a red-dashed rectangle.

2 Supplementary Movie Legends

Movie 1: Wild-type and *spaca6^-/-* sperm are motile. Wild-type or *spaca6^-/-* sperm were imaged 30 seconds after activation using darkfield microscopy. Scale bar = 100 µm. Time: mm:ss.

Movie 2: Wild-type and *spaca6^-/-* sperm can approach the micropyle. Wild-type or *spaca6^-/-* sperm stained with MitoTracker Deep Red (red) were added to wild-type eggs and imaged following sperm addition. Scale bar = 50 µm. Time: mm:ss.

Movie 3: *Spaca6^-/-* sperm are unable to stably bind to wild-type eggs. Wild-type or *spaca6^-/-* sperm stained with MitoTracker Deep Red (red) were added to dechorionated wild-type eggs and imaged following sperm addition. Two minutes after sperm addition, wild-type sperm are stably bound to the egg membrane while *spaca6^-/-* sperm are unable to bind. Scale bar = 50 µm. Time: mm:ss.
